# Adequacy and Effectiveness of Watson For Oncology in the Treatment of Thyroid Carcinoma

**DOI:** 10.3389/fendo.2021.585364

**Published:** 2021-03-03

**Authors:** Hyeok Jun Yun, Hee Jun Kim, Soo Young Kim, Yong Sang Lee, Chi Young Lim, Hang-Seok Chang, Cheong Soo Park

**Affiliations:** ^1^ Department of Surgery, Institute of Refractory Thyroid Cancer, Gangnam Severance Hospital, Yonsei University College of Medicine, Seoul, South Korea; ^2^ Department of Surgery, National Health Insurance Service Ilsan Hospital, Goyang-si, South Korea

**Keywords:** thyroid cancer, Watson for Oncology, IBM, artificial intelligence, treatment recommendation

## Abstract

**Background:**

IBM’s Watson for Oncology (WFO) is an artificial intelligence tool that trains by acquiring data from the Memorial Sloan Kettering Cancer Center and learns from test cases and experts. This study aimed to analyze the adequacy and effectiveness of WFO in determining the treatment method for patients with thyroid carcinoma.

**Materials and Methods:**

We retrospectively enrolled 50 patients with thyroid cancer who underwent surgery in 2018 and entered their clinical data into WFO. The WFO treatment recommendations were compared with the surgical procedures and recommended treatments performed according to the Korean Thyroid Endocrine Surgery Association guidelines.

**Results:**

The overall concordance rate between WFO-recommended treatments and actual surgical treatments was 48%, and for patients with stage I, II, and III disease, these rates were 52.4, 50, and 16.7%, respectively. A lower concordance rate was observed with respect to treatment for advanced thyroid cancer.

**Conclusion:**

WFO is a useful clinical aid but must be used with caution. A surgeon’s decision takes precedence over WFO recommendations in the treatment of advanced thyroid cancer.

## Introduction

Thyroid cancer has a high incidence worldwide. However, thyroid cancer patients have a relatively good prognosis due to a low recurrence rate and high survival rate (1–3). Currently, several recommendations from the American Thyroid Association (ATA), Korean Thyroid Association, and Korean Association of Thyroid and Endocrine Surgeons (KATES) are cited in Korea  (4–6). The contents of these recommendations are comparable to a certain extent.

Watson for Oncology (WFO) (IBM Corporation, United States) is a clinical decision-support system created by IBM and named after its founder, Thomas J. Watson (7). It mainly cites the ATA guidelines to determine the appropriate thyroid cancer treatment. This system uses machine learning to evaluate the clinical factors in a case and determines how treatment options can be identified from the collected data. Its prime function is to recommend the appropriate treatment based on the patient’s electronic medical records. For example, if a patient’s medical records, genetic information, and operability status are entered, WFO recommends a treatment that is verified by oncologists at the Memorial Sloan Kettering Cancer Center (MSKCC). Moreover, certain types of anticancer drugs or combinations thereof and radiation and hormone therapies are also recommended by this system  (8). Additionally, WFO provides evidence to support the credibility of its recommendations, and this evidence is based on relevant literature, such as studies and associated data (9). When a doctor selects a treatment, WFO provides the rate of survival, incidence of side effects, and other related treatment information, helping the doctor assess the overall effectiveness and treatment risk. In agreement with the MSKCC, this technology has provided evidence-based treatment recommendations for cancer patients since 2012 (10), and currently, its applicability extends to various types of cancers such as lung, breast, colorectal, and stomach cancers  (11).

This study aimed to analyze the adequacy and effectiveness of WFO by comparatively analyzing its recommendations with those based on the KATES guidelines.

## Materials and Methods

### Patients

Clinical data from 50 patients diagnosed with thyroid cancer between January 1, 2018, and December 31, 2018, at National Health Insurance Ilsan Hospital were entered into WFO. These patients had undergone surgical treatment following recommendations by the KATES. The data included demographic information such as sex and age; results of medical imaging, nuclear medicine, pathology, and blood testing; and information regarding surgical findings and methods. These data were abstracted from the electronic medical records and entered manually into WFO. This study was approved by the Institutional Review Board of the National Health Insurance Service Ilsan Hospital (IRB No. 2019-01-024).

### Watson For Oncology

WFO treatment recommendations are divided into three groups with corresponding color-coded labels: green indicates “recommended” based on strong evidence; orange indicates “for consideration” implying that oncologists can consider the treatment as an appropriate alternative based on their clinical judgment; and red indicates a “not recommended” treatment due to certain contraindications or lack of strong evidence of use.

### The Korean Association of Thyroid and Endocrine Surgeon (KATES) Guideline

The extent of surgical procedure suitable for differentiated thyroid carcinoma (DTC) is as follows: In the case of micropapillary thyroid cancer of less than 0.5 cm, the surgery timing can determine by selectively providing sufficient information to the patient and observing for a certain period. The initial surgical procedure of patients with DTC of >1 cm is strongly recommended to perform total thyroidectomy or near-total thyroidectomy. It is strongly recommended to perform total thyroidectomy or near-total thyroidectomy for initial surgery in patients with gross extrathyroidal extension, clinical evidence of any lymph node metastasis, or distant metastasis. Thyroid lobectomy is actively recommended for patients with unifocal DTC of <1 cm without a personal history of radiation therapy to the head and neck and familial DTC within the first generation, limited within the thyroid gland, and no clinically detectable cervical nodal metastases.

Lymph node dissection in DTC is as follows: If cervical lymph node metastasis is clinically suspected, therapeutic central compartment lymph node dissection (CCND) should be performed. In the case of T3 or T4 lesions, under 15 years of age or over 45 years of age, multiple lesions, extrathyroidal extension, and lateral cervical lymph node metastasis, prophylactic CCND may be considered even if the central lymph node metastasis is not clinically suspected.

### Concordance

The final comparison target was the surgical treatment suggested by KATES and WFO. Concordance was defined as a match between the surgeon’s treatments that were performed based on recommendations by the KATES  (4) and the WFO’s “recommended” category of surgical treatments. If the surgeon’s treatment was included in the WFO’s “considered” and “not recommended” categories, it was considered a non-concordance.

The null hypothesis was that WFO’s area under the curve (AUC) for the standard treatment is 0.75, while the alternative hypothesis assumed that if the AUC of WFO for the standard treatment is 0.75 or more, the AUC of WFO is 0.95. Therefore, for a type I error of 0.05 and a power of 0.8, the required number of patients would be approximately 40. Further, considering a dropout rate of 20%, 50 participants would be needed to test this hypothesis. As this sample size was achievable within the affiliated institution, the study was not expanded to other research institutes.

## Results

### Patient Characteristics

Among the 50 patients recruited in this study, the majority were women (41, 82%), and the mean tumor size was 0.9 cm. All patients had been diagnosed with papillary thyroid cancer: 32 patients (64%) had bilateral lobe invasion and 29 (58%) had extracapsular involvement. The tumor-node-metastasis stage was classified according to the 8th edition of the cancer staging system by the American Joint Committee on Cancer (12). T1a, T1b, and T2 disease was observed in 32 (64%), 14 (28%), and 4 (8%) patients, respectively; N0, N1a, and N1b disease was observed in 30 (60%), 17 (34%), and 3 (6%) patients, respectively. There were 42 patients (84%) with stage I disease, 2 patients (4%) with stage II disease, and 6 patients (30%) with stage III disease (**Table 1**).

**Table 1:** Patient characteristics.

Characteristics		Total N = 50, No. (%) or mean
Age, year (range)		47.5 (25–77)
Female/Male		41/9 (82%/18%)
Primary tumor size, cm (range)		0.9 (0.3–3.9)
Pathology	Papillary thyroid carcinoma	50 (100%)
Multifocality		32 (64%)
Bilateral involvement		17 (34%)
Extrathyroidal extension		29 (58%)
TNM stage (AJCC 8^th^ edition)		
T stage	T1a	32 (64%)
	T1b	14 (28%)
	T2	4 (8%)
N stage	No	30 (60%)
	N1a	17 (34%)
	N1b	3 (6%)
TNM stage	I	42 (84%)
	II	2 (4%)
	III	6 (12%)

AJCC, American Joint Committee on Cancer; TNM, tumor-node-metastasis.

### Concordance Analyses

**Figure 1:**
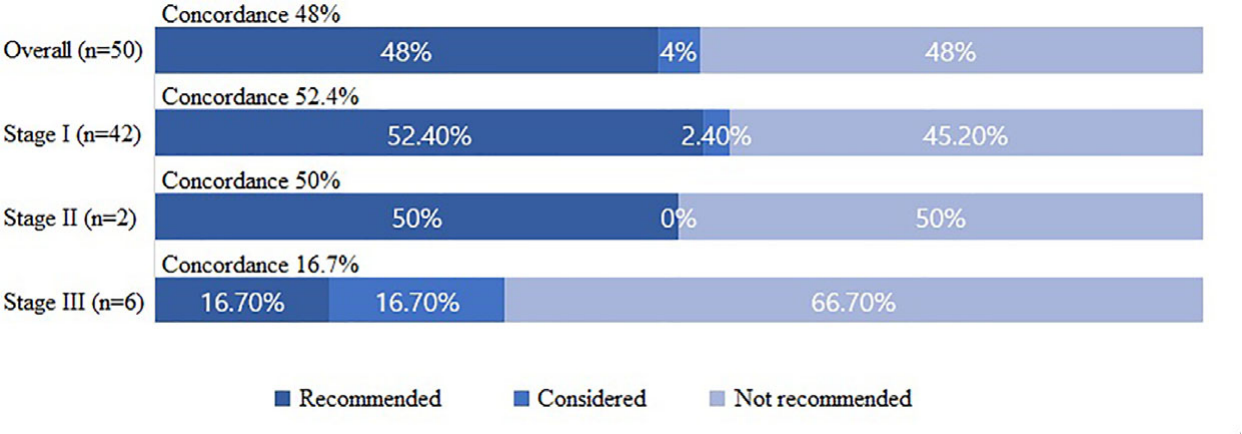
Concordance between the Watson for Oncology-recommended treatment and actual surgical treatment based on the Korean Association of Thyroid and Endocrine Surgeons guidelines.

The concordance rates between the WFO-recommended and KATES-recommended surgical treatment options were analyzed according to the following groups: “recommended,” “for consideration,” and “not recommended.” Overall, the surgical treatments were “recommended” in 24 cases (48%), “for consideration” in 2 cases (4%), and “not recommended” in 24 (48%) cases. The postoperative final results were consistent with the preoperative predictions in all concordant patients. On further analyses, the surgical treatments were “recommended” in 22 cases (52.4%), “for consideration” in 1 case (2.4%), and “not recommended” in 19 cases (45.2%) by WFO among patients with stage I cancers. Among patients with stage II cancer, the surgical treatment was “recommended” in 1 case (50%) and “not recommended” in 1 case (50%). Finally, among patients with stage III cancer, the surgical treatment was “recommended” in one case (16.7%), “for consideration” in 1 case (16.7%), and “not recommended” in four cases (66.7%) by WFO (**Figure 1**).

## Discussion

Artificial intelligence has substantially greater memorization capabilities than the human brain; it can quickly collect and sort the stored information, such as diagnostic radiology applications and applications of pathology imaging systems, to obtain precise conclusions rapidly  (13, 14). WFO was created to serve as a support system that uses vast amounts of medical evidence to aid doctors in treatment planning. Further, it can markedly shorten the time spent by inexperienced surgeons in consulting relevant literature, improving their ability to make accurate diagnoses and prompt treatment recommendations.

In this study, 24 of 50 treatment methods used were consistent with those recommended by WFO, and higher stages of thyroid cancer were associated with a lower concordance rate. Non-concordance between the treatment methods of WFO and KATES is shown in **Table 2**. Non-concordance between the methods considered category by WFO and the KATES was observed in 2 cases. In one non-concordant case, WFO recommends active surveillance, but thyroid lobectomy was performed with careful explanation because the pathological result in repeated fine-needle aspiration was category III of the Bethesda system (15). Final pathological result is papillary thyroid carcinoma. In another case, total thyroidectomy was recommended by WFO because of clinically suspected extrathyroidal extension. But unilateral thyroidectomy was performed because the extrathyroidal extension was not observed during the operation.

**Table 2:** Reason for non-concordance between the WFO-recommended treatment and surgical treatment based on the KATES guidelines.

Reason for non-concordance	WFO recommend treatment	Surgical treatment	Total N = 26
Considered category, n=2			
AUS in repeated FNA	AS	lobectomy	1
ETE	TT	lobectomy	1
Not recommended category, n=24			
Familial thyroid cancer history	AS	lobectomy	1
ETE	TT	lobectomy	7
CCND	TT lobectomy	TT+CCND lobectomy+CCND	16

AUS, atypia of undetermined significance; FNA, fine needle aspiration; AS, active surveillance; ETE, extrathyroidal extension; TT, total thyroidectomy; CCND, central compartment lymph node dissection.

Non-concordance between the methods recommended category by WFO and the KATES was observed in 24 cases. In one non-concordant case, WFO recommended active surveillance and no surgical procedure due to the small tumor size (0.4 cm) and lack of clinical symptoms. However, this patient had undergone unilateral thyroidectomy due to the risk factor of family history (16). An explanation for this non-concordance could be that information regarding risk factors such as family history were not entered in the corresponding data field of the patient’s medical records and examination results.

In 7 patients, total thyroidectomy was recommended by WFO because of clinically suspected extrathyroidal extension. As per ATA and KATES guidelines, thyroid cancer patients with tumors sized > 4 cm or with gross extrathyroidal extension should undergo an initial surgical procedure including a near-total or total thyroidectomy and gross removal of all primary tumors  (5). However, unilateral thyroidectomy was actually performed because extrathyroidal extension was not observed during the operation or on postoperative histopathological examinations. A surgeon with this experience can make appropriate changes to the pre-determined treatment method according to operative findings. However, if the surgeon does not have this experience and relies solely on WFO recommendations for thyroidectomy, it can lead to unnecessary surgery that reduces the patient’s quality of life.

WFO did not recommend central compartment lymph node dissection in 16 patients because no lymph nodes with suspected metastasis were observed preoperatively. However, during the operation, an enlarged central lymph node that indicated metastasis was observed, and a thyroidectomy with central compartment lymph node dissection was performed. Nodal metastasis was confirmed on permanent histopathological examination. In patients with clinically apparent lymph node metastasis, central compartment lymph node dissection improves survival and lowers the risk of recurrence (17). However, in the absence of clinically apparent lymph node metastasis, several guidelines, including those of WFO, do not recommend performing dissection prophylactically (18, 19). Therefore, inexperienced surgeons solely relying on WFO recommendations may inadvertently neglect metastatic lymph nodes.

From another perspective, confirming seen in preoperative imaging studies (CT, US) is essential during surgery. However, image studies cannot completely determine whether there is extrathyroidal extension or lymph node metastasis. Therefore, Non-concordance in determining the extent of surgery due to extrathyroidal extension or lymph node metastasis may be due to imaging studies’ limitations, not the WFO itself.

In 6 stage III patients, 5 showed non-concordance. The average age of 5 patients was 57.5 years, four patients with T1b and 1 with T2 each, and all patients with N1a. there were no particular differences from other stage except age. Four cases showed non-concordance depending on whether CCND was performed or not. In another case, WFO recommends active surveillance, but thyroid lobectomy was performed.

Evidence-based medicine forms the backbone of modern medicine and entails that patients be treated according to a clear medical rationale, unlike that in the past, when the treatment heavily relied on the intuition or experience of the doctor. WFO is a technology that uses a clear medical rationale and has the capability to learn and recall all of the abundant and constantly updated evidence. Nonetheless, the planned treatment or surgical extent may change according to intraoperative findings in some cases, reinstating the importance of the surgeon’s judgment.

There are several limitations to our study. First, it was a retrospective observational study that lacked controls; this made the results potentially vulnerable to the influence of unmeasured factors. Second, the group of patients with stage I cancer was considerably larger than the other groups. This study is a preliminary study, conducted only in a group of 50 patients. In the clinical field, there are more patients in stage I than stage II and stage III, but the results will be more accurate if more patients are studied in the future. Third, the results may vary if different versions of WFO or the KATES guidelines are used. Updated versions of the these WFO and KATES guidelines should be validated in further research.

In conclusion, WFO is useful as an assistive tool for doctors. However, it must be used with caution because the WFO recommended treatment for patients with advanced thyroid cancer may not be the most appropriate approach. Therefore, the surgeon’s judgment gains precedence over WFO recommendations in determining the treatment method for patients with advanced thyroid cancers. In the future, a multicenter study should be conducted to investigate the adequacy and Effectiveness of WFO in the treatment of thyroid carcinoma.

## Data Availability Statement

The raw data supporting the conclusions of this article will be made available by the authors, without undue reservation.

## Ethics Statement

The studies involving human participants were reviewed and approved by Institutional Review Board of the National Health Insurance Service Ilsan Hospital (IRB No. 2019-01-024). Written informed consent for participation was not required for this study in accordance with the national legislation and the institutional requirements.

## Author Contributions

Study concept and design: CL and YL. Acquisition, analysis, or interpretation of data: HY, HK, SK, HC, and CP. Drafting of the manuscript: HY, CL, and YL. Statistical analysis: HY and CL. All authors contributed to the article and approved the submitted version.

## Funding

This research was supported with research funds given by the National Health Insurance Service, Ilsan Hospital (NHIMC2019CR014).

## Conflict of Interest

The authors declare that the research was conducted in the absence of any commercial or financial relationships that could be construed as a potential conflict of interest.

## Abbreviations

ATA, American Thyroid Association; AUC, Area under the curve; KATES, Korean Association of Thyroid and Endocrine Surgeons; MSKCC, Memorial Sloan Kettering Cancer Center; WFO, Watson for Oncology.
